# Initial Morphology and Feedback Effects on Laser-Induced Periodic Nanostructuring of Thin-Film Metallic Glasses

**DOI:** 10.3390/nano11051076

**Published:** 2021-04-22

**Authors:** Mathilde Prudent, Florent Bourquard, Alejandro Borroto, Jean-François Pierson, Florence Garrelie, Jean-Philippe Colombier

**Affiliations:** 1 Univ Lyon, UJM-Saint-Etienne, CNRS, Institute of Optics Graduate School, Laboratoire Hubert Curien UMR CNRS 5516, F-42023 St-Etienne, France; mathilde.prudent@univ-st-etienne.fr (M.P.); florent.bourquard@univ-st-etienne.fr (F.B.); florence.garrelie@univ-st-etienne.fr (F.G.); 2 Université de Lorraine, CNRS, IJL, F-54000 Nancy, France; alejandro.borroto@univ-lorraine.fr (A.B.); jean-francois.pierson@univ-lorraine.fr (J.-F.P.)

**Keywords:** thin film metallic glasses, femtosecond laser, LIPSS, surface functionalization, nanostructuring

## Abstract

Surface nanostructuring by femtosecond laser is an efficient way to manipulate surface topography, creating advanced functionalities of irradiated materials. Thin-film metallic glasses obtained by physical vapor deposition exhibit microstructures free from grain boundaries, crystallites and dislocations but also characterized by a nanometric surface roughness. These singular properties make them more resilient to other metals to form laser-induced nanopatterns. Here we investigate the morphological response of Zr_65_Cu_35_ alloys under ultrafast irradiation with multipulse feedback. We experimentally demonstrate that the initial columnar microstructure affects the surface topography evolution and conditions the required energy dose to reach desired structures in the nanoscale domain. Double pulses femtosecond laser irradiation is also shown to be an efficient strategy to force materials to form uniform nanostructures even when their thermomechanical properties have a poor predisposition to generate them.

## 1. Introduction

For several decades, the femtosecond laser has been considered as a universal one-step procedure allowing the functionalization of solid materials. Benefitting from a high flexibility, ultrashort laser-induced surface nanostructuring has a panel of applications in various domains such as biomedical technologies, nanofluidics, renewable energies or aeronautics, to name a few [1,2,3]. The preparation and structuring of material surfaces on the nanometer scale are of prime importance for the advancement of these applications. The fast and repeated cycles of melting and resolidification upon multiple pulse irradiation progressively build up unique surface morphologies. Main topography features can be dictated by tuning the laser wavelength, polarization states, pulse duration or the fluence conditions, able to statistically promote specific shapes, scales, depths and orientations. These structures can transform or enhance surface properties such as wettability [4,5], mechanical and corrosion behavior, optical properties [3] or tribological performances [6], giving new functionalities to the structured material. More specifically, laser-induced periodic surface structures (LIPSS) with various characteristics can be generated on transparent materials, semi-conductors or metals [7,8]. For laser–metal interaction, several class of micro- and nano-structures are classically encountered. Formed near the ablation fluence regime, low spatial frequency LIPSS (LSFL) exhibit a periodicity marginally lower than the laser wavelength *λ*. LSFL are perpendicular to the electric field polarization, and are triggered by the interference of the incident light with the radiative scattered fields from laser-induced surface heterogeneities as bumps, holes or nanoparticles. Parallel or perpendicular to the laser polarization, high spatial frequency LIPSS (HSFL) have a period lower than *λ*/2 and are strongly suspected to be initiated by local field enhancement on nanoreliefs whereas their growth is sustained by hydrodynamic mechanisms [9]. They are often categorized according to features depending on their localization and their orientation parallel or perpendicular to the polarization [10]. Parallel to the polarization and with a periodicity *Λ* > *λ*, a groove-like structures can also be generated on metals by high feedback femtosecond laser irradiation [11]. Finally, nanocavities or nanobumps were recently disclosed on the nanoscale with original hexagonal symmetries [9,12,13,14].

Even if high regularity process has been reported [15], typical LSFL generated on metals used to present many heterogeneities and bifurcations, limiting the process to be competitive for advanced industrial applications as nanolithography. Contrary to the crystalline metals, bulk amorphous metals, also named bulk metallic glasses, have the particularity to form very regular LSFL with few bifurcations [16]. This may be due to the excellent surface state of these amorphous materials, free from any topographic inhomogeneities. Indeed, the absence of crystalline defects such as grain boundaries or dislocations reduces the roughness and the number of surface heterogeneities [15]. However, few works deal with the HSFL creation on amorphous metals, limiting the main structures to LSFL and rings [16,17,18,19]. Although these bulk metallic glasses are already used in niche markets, they are very expensive to fabricate and, since the 1980s, thin-film metallic glasses (TFMG) have been elaborated varying the composition systems. The physical vapour deposition (PVD) method, and in particular magnetron sputtering, constitutes one of the most popular elaboration techniques, allowing the control of the composition, the density and the morphological aspect of the films. Zirconium-based TFMG can considerably increase the mechanical properties and the tribological behaviour of the covered material. Thereby, a Zr-Cu system constitutes a TFMG thoroughly studied because of its good properties and also for its good glass forming ability [20].

The aim of this work is to exploit the special structural nature of Zr-Cu thin film to investigate the susceptibility of this metallic glass to form nanopatterns exhibiting periods down to 100 nm. To understand the mechanisms underlying their emergence, we have investigated the effects of different initial morphologies. Two TFMG with the same composition and different surface aspects were irradiated varying the irradiation conditions. The difference in terms of feedback was revealed by electron and atomic microscopies.

## 2. Material Surface Features and Ultrafast Irradiation Process

Two thin films of metallic glass with the composition Zr_65_Cu_35_ have been deposited for the experiments. They were both manufactured by PVD magnetron sputtering from two targets of zirconium and copper. The thin films were synthesized on a silicon wafer. The argon working pressure was modified between the two samples, inducing two different kinds of film morphology [20,21]. The first sample, named “Tight Columns” (TC sample), was manufactured with a working pressure of 1 Pa while the second one, named “Coarse Columns” (CC sample), was deposited under 0.5 Pa. The substrates were not intentionally heated during the film deposition and the sample temperature was lower than 80 °C. The surface morphologies were observed by scanning electron microscopy (“SEM”, Nova NanoSEM 200, FEI, Hillsboro, OR, USA) and atomic force microscopy (“AFM”, Dimension ICON, Bruker, Billerica, MA, USA) in ScanAsyst-Air mode with a silicon nitride probe, coupled with image analysis. These analyses are described in part 3.

To perform periodic structuring of the surface, femtosecond laser irradiations were carried out using a commercial Ti: sapphire laser system (Legend Elite, Coherent Inc. San Jose, US), producing pulses centered at *λ* = 800 nm wavelength with a pulse duration fixed at 60 fs. The beam was focused on the samples by a 25 cm achromatic lens. Double pulses with the possibility to adjust the delay between them were produced by splitting and recombining the beam using a Mach–Zehnder interferometer configuration with a mobile arm. In each optical path the combination of a half-wave plate and a polarizer allowed for a perfect control of the pulses polarizations and energies [14]

**Figure 1:**
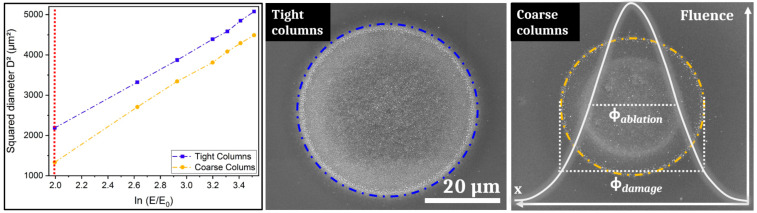
Evolution of the squared diameter versus the neperian logarithm of the energy of irradiation for a single pulse irradiation. The scanning electron microscope (SEM) images show the impact aspect for both samples for the lowest irradiation fluence (F_1p_ = 0.25 J/cm^2^) corresponding to the value displayed by the red line: ln(E/E_0_) = 2 (with E_0_ = 1 µJ).

Using the D^2^ technique [22], the theoretical spot size and the damage threshold fluence for 1 laser pulse were determined for both samples. Figure 1 shows the evolution of the squared diameter increasing the irradiation energies for both samples for one single shot. Figure 1 also displays two SEM pictures of the impacts after one pulse obtained after irradiation for a fluence of 0.25 J/cm^2^. The diameters of the spots were determined by an accurate measurement of the observed damage area. The D^2^ technique yields the damage threshold fluences for one laser pulse for both samples. The TC sample presents a threshold fluence of F_th-TC_ = 0.08 J/cm^2^ lower than those of the CC sample with F_th-CC_ = 0.12 J/cm^2^.

For single pulse experiments, different fluence conditions of irradiation were tested on both samples and three of them are reported and analyzed in the paper: 0.12, 0.08 and 0.06 J/cm^2^. Different numbers of laser pulses were investigated from 1 to 50 pulses. For double pulse experiments, the polarizations of both pulses were horizontal and the lowest fluence condition (0.06 J/cm^2^) is shown here. The burst number was fixed to 50 pulses and the time delay between the two pulses was increased from 0 ps to 70 ps. All of the irradiations were performed at atmospheric pressure in ambient air.

## 3. Experimental Results

### 3.1. Single Pulse Laser-Induced Nanostructuring: Initial Morphology Dependence on the Feedback

**Figure 2:**
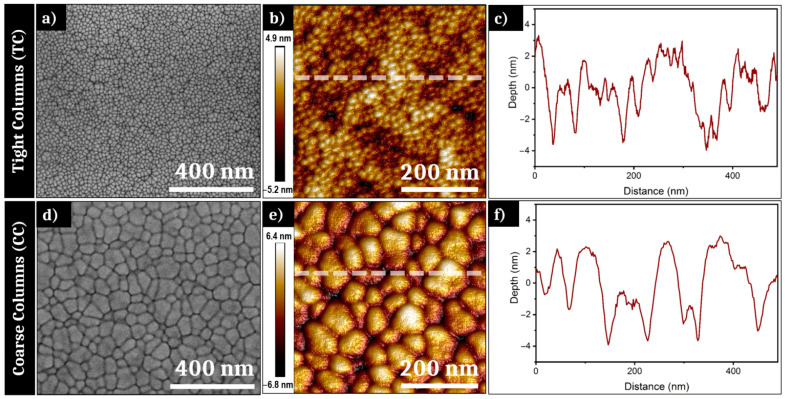
(**a**) Scanning electron microscope (SEM) picture and (**b**) atomic force microscope (AFM) 3D-projection of the surface of the Tight Columns (TC) sample; (**c**) height profile of the white line in (**b**); (**d**) SEM picture and (**e**) AFM 3D-projection of the surface of the Coarse Columns (CC) sample; (**f**) height profile of the white line in (**e**).

As mentioned in part 2, both samples used for the irradiation experiments have different initial morphologies resulting from different pressure conditions during PVD elaboration. Figure 2a,d show SEM pictures of the surface of, respectively, the TC and CC samples. The surface morphology of both samples displays a high density of interstices between so-called columns, which are classically formed during the PVD process. In an interesting way, the grain size observed on the surface plane varies with the pressure conditions. The TC sample presents a “granular” morphology with a size distribution denser and more regular than the CC sample. An image analysis reveals that the transverse size of the TC columns vary between ~10 and ~50 nm of diameter. The CC sample exhibits larger columns on the surface, with a typical width between ~30 and ~140 nm of diameter. If SEM provides high-quality images of the prepared surfaces, this remains limited to provide quantitative information of those morphologies and require complementary microscopy to define precise roughness profiles.

To complete the information visible on the SEM pictures, AFM measurements were carried out for both samples. Figure 2b,c respectively present an AFM analysis of the surface of the Tight Columns sample and a height profile of the white line visible on Figure 2b. The “peak-to-peak” roughness of the TC sample is measured at 10.1 nm, for an average RMS (root mean square) roughness of 1.44 nm. This profile shows a low regular distribution and confirms the average column’s size found with the image analysis. Figure 2e,f display the AFM analysis of the initial morphology surface of the CC sample and the height profile of the white line displayed on the AFM picture. The roughness range is marginally higher than for TC as the measure gives a “peak-to-peak” roughness of 13.2 nm and a RMS roughness of 1.98 nm for CC. The height profile reveals an average column size in accordance with the observations done on the SEM picture. Figure 2f also shows small bumps on the top of many large columns, confirming the heterogeneity of roughness distribution for this sample. Thereby, both samples exhibit very low roughness, with optical surfaces of high quality with irregularities of the dimension <*λ*/100.

**Figure 3:**
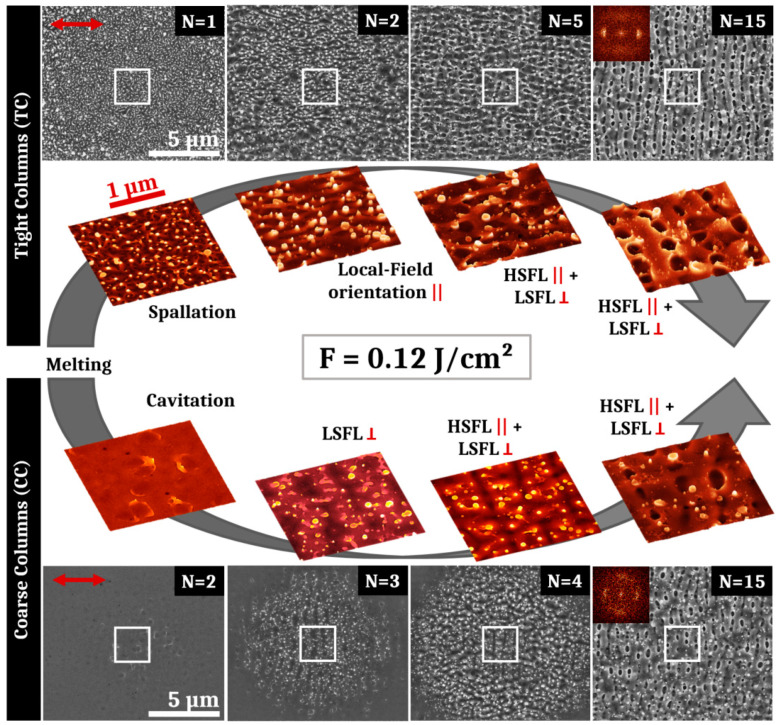
SEM and 3D-projections of SEM pictures of surface structures obtained after irradiation of the Tight Columns and the Coarse Columns samples with an average fluence of 0.12 J/cm^2^ and different numbers of pulses. The evolution of structures is shown for different numbers of pulses. For the TC sample, successively appear disorganized filaments induced by spallation (N = 1), orientated filaments (N = 2), high spatial frequency laser-induced periodic surface structures (HSFL) parallel to the polarization intertwined between low spatial frequency laser-induced periodic surface structures (LSFL) perpendicular to the polarization (N = 5 and N =15). For the CC sample, successively appear residual cavitation bubbles (N = 2), LSFL (N = 3), horizontal HSFL and vertical LSFL (N = 4 and N = 15). The red arrow represents the electric field polarization. The symbols (||) and (ꓕ) respectively mean “parallel” and “perpendicular” to the polarization. 2D-Fourier transforms shown as insets support the presence of a crossed structure of HSFL and LSFL for the highest pulse numbers.

The first laser experiments were done with a fluence of 0.12 J/cm^2^ corresponding to the CC sample’s one pulse damage threshold, which is higher than that of the TC sample. After the irradiations with an average fluence of 0.12 J/cm^2^, LSFL are obtained for high numbers of laser pulses for both samples. Figure 3 presents the SEM pictures corresponding to the evolution of structures obtained on both samples for different pulse numbers N. The red arrow shows the electric field polarization. Colored 3D-projections of SEM images correspond to the white box visible on each large SEM pictures.

Regarding the evolution of structures for the TC sample, it is remarkable that for the first pulse, frozen liquid bridges are already present on the impact. They are characteristic of the spallation mechanism. After two pulses, these filaments orientate preferentially in parallel with the laser polarization direction. They are observable up to 4 pulses and, for a higher feedback, nascent HSFL are oriented along this preferred direction. However, HSFL are superposed to underlying LSFL, revealing a crossed structure that mixes both types on the center of the impact. LSFL are clearly present from 15 to 50 pulses with HSFL perpendicular to them and mostly visible in the hollows. The irradiation fluence used in this case (0.12 J/cm^2^) is 50% more elevated than the damage threshold of the TC sample. The thin film experienced a photomechanical ablation regime with identifiable specificities as surface structures revealed on Figure 3. After heating, the amorphous material turns to a molten state that affects and partially erases the initial columnar morphology. During this liquid state, spallation induced by generation and coalescence of voids in the liquid layer generates filaments that remain on the surface after the solidification process [23]. Near-field localized in the cavities between the filaments lead to the apparition of HSFL parallel to the electric field direction. Resulting from far-field scattering on the roughened surface, LSFL start to appear at the same time perpendicularly to the HSFL and their definition increase for high numbers of pulses [7,9,24].

For the Coarse Columns sample irradiated by one single shot at 0.12 J/cm^2^ (not presented on Figure 3), the laser impact is barely visible on the surface and no structure can be evidenced. However, the initial columnar surface morphology disappears. For N = 2, structures resulting from exploded cavitation bubbles are visible on the surface. For N = 3, periodic structures perpendicular to the polarization are already discernible at the impact centre. They present a period marginally lower than the wavelength of the laser, and constitute the precursor of low spatial frequency LIPSS. From 4 to 10 pulses, HSFL formation, parallel to the electric field, emerge between the LSFL structures. Increasing the number of pulses, LSFL are increasingly defined. The definitive crossed structure with mixed HSFL and LSFL is observable on Figure 3 for 15 pulses for the CC sample. This crossed structure is present up to 50 pulses.

Although the final crossed structures mixing HSFL and LSFL are similar for both samples, the structure evolution differs between the CC sample and the TC sample. The average roughness for both samples is very low (less than 2 nm), but the different roughness morphology affects considerably the damage threshold fluence leading to a delayed surface structures apparition and evolution in terms of feedback. For the CC sample, after the surface melting stage, a cavitation mechanism occurs leading to the apparition of surface bubbles but spallation filaments are less pronounced than for the TC sample. Large cavitation bubbles appear with a low density and constitute larger precursors than the filaments for light scattering compared to the TC sample. These frozen cavitation structures trigger the apparition of low spatial frequency LIPSS perpendicular to the electric field. For a higher number of pulses, the LSFL hollows undergo local melt flow that initiates high spatial frequency LIPSS parallel to the polarization [9]. While they are less regular than for the TC sample, LSFL contrast increases for higher number of pulses. For N = 15 pulses, the period of the LSFL on the TC sample is ~630 nm versus ~650 nm for the LSFL of the CC sample. Comparing LIPSS on this TFMG with that already reported on bulk metallic glass, the LSFL obtained here present many heterogeneities, bifurcations and redeposited particles [25,26]. As they are triggered by sharp nanoreliefs in the photomechanical regime of ablation, the HSFL present also bifurcations and irregularities for both samples [16,17]. Moreover, in both cases, HSFL are superposed with LSFL structures. To favor the HSFL apparition and prevent the formation of LSFL, the fluence has been reduced to 0.08 J/cm^2^.

**Figure 4:**
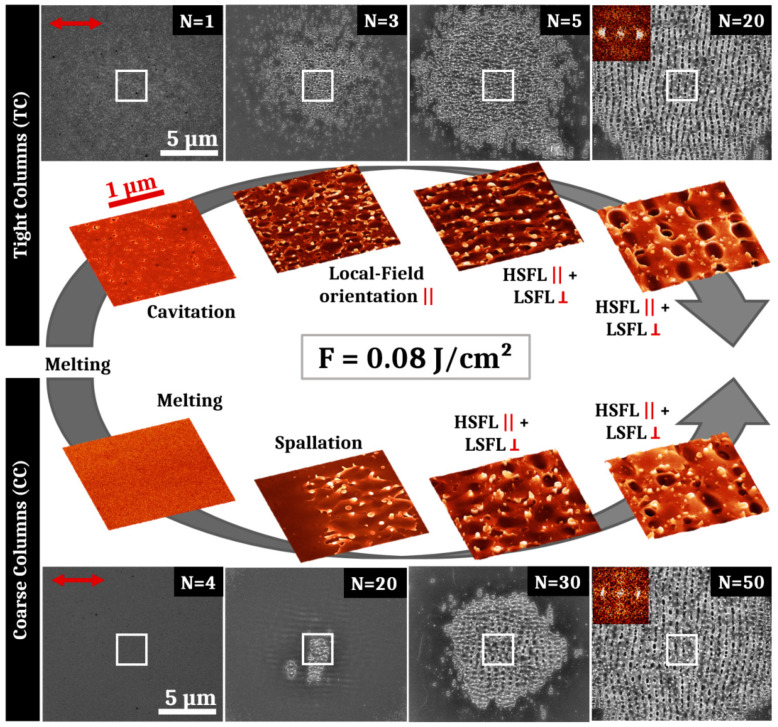
SEM and 3D-projections of SEM pictures of surface structures obtained after irradiation of both samples with an average fluence of 0.08 J/cm^2^ and different numbers of pulses. The evolution of structures is shown for different numbers of pulses. For the TC sample, successively appear residual cavitation bubbles (N = 1), disorganized filaments (N = 3), HSFL parallel to the polarization intertwined between LSFL perpendicular to the polarization (N = 5 and N = 20). For the CC sample, successively appear a smooth surface (N = 4), spallation effects (N = 20), horizontal HSFL and vertical LSFL (N = 30 and N = 50). The red arrow represents the electric field polarization. The symbols (||) and (ꓕ) respectively mean “parallel” and “perpendicular” to the polarization. 2D-Fourier transforms shown as insets support the presence of a crossed structure of HSFL and LSFL for the highest pulse numbers.

The results of these second irradiations performed at a lower fluence of 0.08 J/cm^2^ are presented in Figure 4, showing the surface structures obtained on both samples after different pulse numbers. Similar periodic surfaces structures are visible for the TC sample in order of appearance: oriented filaments, HSFL and LSFL. However, at this reduced fluence, spallation effects are not visible for N = 1 and the material surface has undergone a localized cavitation process with a high concentration. These surface cavities are not leading to the apparition of LSFL but the smaller scattering centers have stimulated localized field enhancement that orientates filaments along the electric field direction. For the CC thin film, no surface structure is visible for N < 10 pulses as the fluence was lower than the damage threshold of the sample. At 10 pulses, structures parallel to the polarization with a period similar as LSFL are present with a very low contrast. On the top of these structures, some cracks seem to be the precursors of spallation effects, visible for N > 20. These cracks may appear in response of the particular deformation modes of metallic glasses that generate high stresses [20,27]. After spallation mechanisms (N = 20), HSFL mixed with LSFL appear for N = 30 pulses. Finally, for higher numbers of pulses, LSFL are observable on the CC sample with a better contrast and more regularly than for the higher fluence.

For both fluences (0.12 J/cm^2^ and 0.08 J/cm^2^), HSFL are obtained concurrently with LSFL. These two types of structure are always superposed with a better definition of either one or the other depending on the pulse number. The HSFL formed are always less regular than those reported on crystalline metals [28,29]. In order to create homogeneous and regular high spatial frequency LIPSS, irradiations in a sub-ablation regime were performed with a fluence lower, at 0.06 J/cm^2^, and increasing the feedback.

### 3.2. High Feedback with Single and Double Pulses Strategy to Foster High Spatial Frequency Laser-Induced Periodic Surface Structures (HSFL)

**Figure 5:**
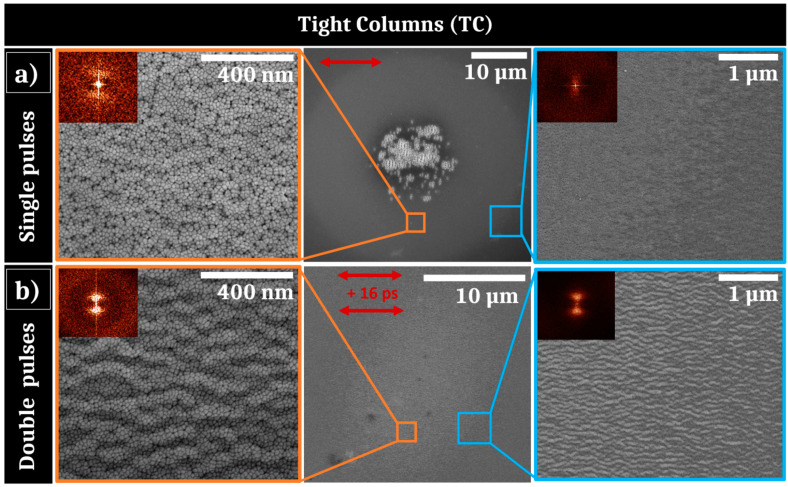
(**a**) SEM pictures of irradiated area of the Tight Columns sample after 50 single pulses of laser irradiation with 0.06 J/cm^2^ with 2D-Fourier transform as insets; (**b**) SEM pictures of irradiated area after 50 double pulses of laser irradiation separated by 16 ps with a total fluence of 0.06 J/cm^2^ with 2D-Fourier transform as insets. The red arrows represent the electric field polarizations.

Using a low fluence of 0.06 J/cm^2^ with single pulses experiments, the samples are submitted to a sub-ablation regime promoting solid–liquid phase transition that may involve hydrodynamic instabilities due to a capillary process [9]. These instabilities are correlated to time-dependent liquid properties that can be extended using a collinear double pulses approach [9]. Therefore, a second set of irradiations were done at the same fluence of 0.06 J/cm^2^ in a double pulse configuration, with an equally distributed fluence in each arm, with an elevated number of pulses in order to enhance the feedback. Figure 5 shows comparatively the results obtained for both 50 single-pulse and 50 double-pulse sequences. For single pulses (Figure 5a), the TC sample exhibits a reduced zone where low spatial frequency LIPSS are formed at the center of the irradiation area. Two zooms at remote locations surrounding the LSFL are presented in the Figure 5a (left and right) with their respective 2D-Fourier transform (2D-FT) as insets. On the border of the observed laser impact area, weakly contrasted nanostructures are present. They consist of periodic arrangement of material, parallel to the polarization, with a period approximately equal to 100 nm. These periodic structures are only observed at the outside region of the Gaussian energy distribution and their contrast is similar to structures associated to annealing mechanisms [13,30]. In addition, in the intermediate region between these structures and the impact center, highly-concentrated dark contrasted points are visible at the interstices of the initial columnar morphology. These dark points indicate the presence of cavitation-induced gaps between the columns. Low contrasted wavy patterns, confirmed by vertical bright dots in the 2D-Fourier transform, reveal the starting of HSFL generation that preserve the microstructural morphology.

Figure 5b displays the results obtained in a similar energy dose but with a different fluence feedthrough brought by two consecutive pulses, delayed by a time Δt=16 ps. This corresponds to the optimal time separation between both horizontally polarized pulses to form HSFL. These nanostructures are, however, observed in the range Δt=2−30 ps, with a maximum contrast at Δt=16 ps. For this delay condition, the impact is homogeneously covered by this original kind of HSFL and two different magnitude of zooms are presented at the border of the impact in Figure 5b with the associated 2D-FT as insets. The distributed HSFL are noticeably regular and present a period Λ~100 nm at the center and also at the border of the laser impact. They present an amplitude between 15 and 20 nm. Once again, the persistence of grains is remarkable and questions on the thermodynamic conditions underlying this phenomenon.

**Figure 6:**
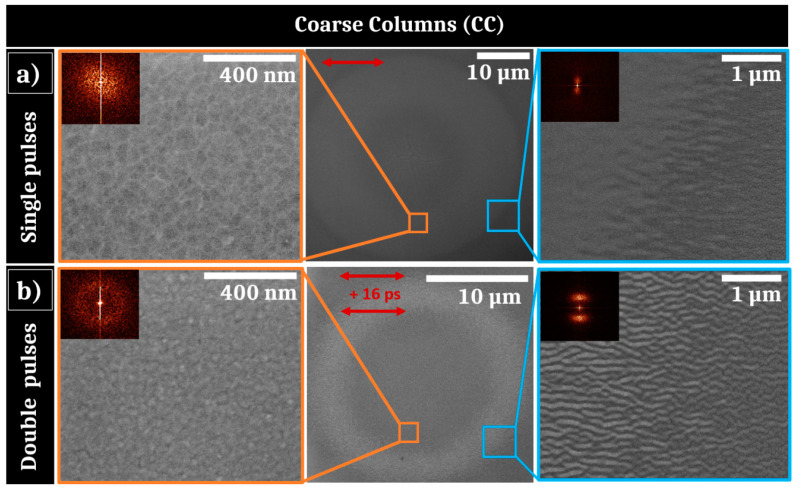
(**a**) SEM pictures of irradiated area of the Coarse Columns sample after 50 single pulses of laser irradiation with 0.06 J/cm^2^ with 2D-Fourier transform as insets; (**b**) SEM pictures of irradiated area after 50 double pulses of laser irradiation separated by 16 ps with a total fluence of 0.06 J/cm^2^ with 2D-Fourier transform as insets. The red arrows represent the electric field polarizations.

The same irradiations at 50 single- and double-pulse sequences were applied to the CC sample at 0.06 J/cm^2^. These results are shown on Figure 6. As previously noted for the TC sample, for single pulse irradiation shown in the middle of Figure 6a, the border of the irradiated zone of the CC sample presents horizontally periodic surface structures that may be generated by an annealing process. However, these formed structures are larger than those observed on the TC sample, that may be due to the larger size of coarse columns or to a different thickness of heated/liquid layer. On the center of the impact, LSFL are not observed as for the TC sample, indicating that the energetic dose condition is not sufficient to initiate them. Instead, some big cracks are present at the center of the Gaussian fluence distribution, probably resulting from intense tensile stresses at the surface of the thin-film metallic glass. Around these cracks, a SEM zoomed picture reveals that the initial columnar morphology of the TFMG has disappeared. The interstices are less contrasted than before irradiation and the roughness is likely to be lower. The 2D-Fourier analysis performed in this irradiated region confirms the absence of order.

For the double pulse experiments presented in Figure 6b (middle), the material response is not as homogeneous as that observed for the TC sample and a bright corona with a 10 µm radius is visible at the inner edge of the laser impact. A zoomed picture of this zone (Figure 6b right) reveals the presence of high spatial frequency LIPSS, parallel to the polarizations. The impacts are similar for time delays Δt=2−30 ps with more contrasted HSFL at Δt=16 ps, as observed for the TC sample. The HSFL have a period *Λ*~150 nm, slightly smaller than the periodic structures created on the border of the single-pulse impact visible on Figure 6a. Furthermore, they are more contrasted, as evidenced on the SEM picture, that is confirmed by the 2D-FT. The center zone does not exhibit HSFL and the “columnar” morphology of the film has disappeared as well as for single pulse experiments; 2D-FT analysis validates the absence of periodic structures near the center (Figure 6b left).

As a resume, a double-pulse sequence enhances the regularity and contrast of HSFL formation. This has been optimized by a specific time delay between the two collinear pulses that could correspond to the thermomechanical characteristic time of the ultrafast irradiated surface. They emerge as the material is heated near the melting threshold, enabling the preservation of the initial columnar morphology for the TC sample, but counter-intuitively, the initial columns for the CC sample disappear. Even if the one-pulse damage threshold is lower for TC, this may not the case for a higher number of shots for which the CC sample damage threshold could be lower. Thereby, this confirms that the feedback dynamics is strongly morphology-dependent.

## 4. Discussion

Ultrashort-laser irradiation of Zr_65_Cu_35_ thin film metallic glass shows that standard LIPSS formation can be achieved with a fluence adjusted to the initial morphology. In particular HSFL and LSFL with polarization-driven orientations propose features similar to what is usually observed in crystalline metals [8,31,32]. It is important to point out that behind the generic term of HSFL, nanostructures show two kinds of very distinct morphologies for situations where spallation process occur on the surface, as shown on conditions of Figure 3 and Figure 4, and when the surface seems to conserve its general integrity, in particular conserving columnar growth patterns as observed for Figure 5 and Figure 6. 

Both kinds of PVD prepared thin film, with distinct roughness shown in Figure 2, allow progressively the establishment of LSFL when the number of laser shots increases as topography-driven feedback offers all the requirements for surface waves scattering [33]. This is expected in the classic scenario stipulating an interference process between the incident pulse with the surface scattered waves [34]. SEM images of the impact after tens of impacts, presented in Figure 3 and Figure 4 for two different fluences, confirm that the conditions required to form LSFL are poorly correlated to the initial roughness even though we need to adjust energetic dose conditions. This observation was anticipated as the coherent far-field scattering is weakly dependent on the topography morphologies, supporting the “surface-scattered wave” as a robust concept not very demanding on the initial conditions [34,35]. More interestingly, the transient evolution of both surfaces is slightly different and the CC sample exhibits delayed dynamics in terms of feedback or requires a higher fluence to achieve LSFL, in accordance with the threshold of material response. We can note that this different response between both initial morphologies subsides as the number of pulses increase, i.e., that the cumulative dynamics differ.

At 0.12 J/cm^2^ and 0.08 J/cm^2^ fluences, the photomechanical regime of ablation is gradually set up. This induces a spallation process with coalescence of sub-surface voids associated with breakup and fast freezing of the transient liquid structures remaining on the surface [23]. The retraction dynamics of liquid droplets, driven by capillary and viscosity forces, play a key role for the formation of further reliefs at the nanoscale. Pulse after pulse, the progressively formed roughness centers experience non-radiative light coupling that will trigger orientation of energy deposition and transverse thermal gradients. The polarization-oriented structures around asperities are clearly visible on Figure 3. A high concentration can favor multiple scattering whereas the size and shape of a relief unit determines the strength of the local field enhancement. This complexity and diversity of nanoreliefs prevent or limit the effective control of this kind of HSFL. Moreover, initiating and associating with an ablation regime, these structures are superposed to more resilient LSFL, which lessen the potential properties of this kind of HSFL. In our irradiating strategy, a particular emphasis was thereby placed on the apparition of uniform nanostructures well below the wavelength, parallel to the laser polarization, and not superposed to LSFL. This requires the fluence to decrease from 0.12 J/cm^2^ to 0.06 J/cm^2^ whereas the number of pulses has to increase as revealed by SEM images shown in Figure 3, Figure 5 and Figure 6. In absolute terms, as previously reported on other materials, a high feedback regime for fluence conditions close to melting threshold fosters the emergence of wavy structures with structural modifications combined with a periodic topography while limiting the flatness alteration of the thin film [13,36]. This point is crucial for targeted mechanical and tribological applications.

Finally, in a sub-ablation regime, hydrothermal waves driving the hotter melt flow above the initial surface level were shown to be at the origin of HSFL growth for metals and semi-conductors [9,13]. Regular nanopatterns inherit the light polarization response on the local roughness with a formation resulting from the competition between destabilizing gradients as surface tension and a rarefaction wave and thermal dissipation on the surface. In this Bénard–Rayleigh–Marangoni-like hydrodynamic instability resides the lifetime of the molten layer as well as an efficient displacement of the liquid interface [9]. This suggests that the viscous flow is facilitated by the non-equilibrium properties of the system. In particular, the related characteristic time for Marangoni flow against the viscous forces is estimated as $\tau_{M}=\frac{{\mu L}^{2}}{4h\gamma \Delta T}$ where μ is the material viscosity, L the expected distance, h the liquid thickness and γΔT the surface tension dependence. At the melting temperature estimated to $T_{m}=1300$ K, the viscosity is around 50 mPa s which is one order of magnitude higher than the values reported for most crystalline metals [37]. However, at higher liquid temperatures in the range T= 2000–2500 K, μ decreases to values similar to pure metals and reaches 2–6 mPa s, enabling a strongly more pronounced capillary-driven melted flow [37]. Reducing this Marangoni timescale, the liquid-displacement from hot to cold regions can modulate surface topography before thermal dissipation and solidification. To exploit this high-T dependence of viscosity for amorphous metals, a collinear double pulse irradiation strategy has been followed varying the pulse delay. This way, the material is brought to a higher temperature as long as possible with the opportunity to control cavitation formation by the time separation between two pulses. Figure 5 and Figure 6 clearly show that the double-pulse sequence promotes HSFL formation preventing LSFL apparition at the low fluence regime. Double-pulse irradiation provides this way an additional degree for controlling the desired material properties far from equilibrium. This easily implementable beam engineering opens the route to functionalize materials, as thin film amorphous metallic alloys, unwilling to form uniform HSFL.

## 5. Conclusions

Since Sipe’s theory, it has been clearly established that the roughness layer has a central role in the formation of LIPSS, in particular for LSFL development. Thin film deposition by PVD offers a remarkable way to change the initial morphologies conserving other alloy properties. In particular, high-quality surface metallic glass exhibits granular columns that can affect the transient ultrafast light coupling under multipulse feedback irradiation. Although the initial average roughness of the film is very low, the columnar morphology constitutes roughness heterogeneities inducing more bifurcations than for polished bulk amorphous metals in laser-induced surface structures. These heterogeneities can be comparable to crystalline defects as grain boundaries or dislocations and represent privileged precursors for local light absorption and scattering. This is a valuable asset to favor non-radiative field structures and subsequent HSFL creation despite the resilience of metallic glass to form such nanopatterns. We show that the high viscosity of an amorphous alloy can be bypassed by double-pulse irradiation sequences that bring the material into a higher temperature and a presumed lower viscosity state more favorable to undergo required capillary flows. This temporal optimization of the energy feedthrough promotes the development of homogeneous high spatial frequency LIPSS with a high regularity and preserving the surface integrity. Therefore, ultrashort laser pulses can functionalize thin-film metallic glasses generating patterns with various periodicities, down to 100 nanometers. These results pave the way for the use of a femtosecond laser process for nanostructuring and further functionalization of metallic glass thin film, with dimensions and accuracies relevant for mechanical, tribological and biomedical applications.

## Data Availability

Not applicable.

## References

[1] Bonse J., Höhm S., Kirner S.V., Rosenfeld A., Krüger J. (2017). Laser-Induced Periodic Surface Structures—A Scientific Evergreen. IEEE J. Sel. Top. Quantum Electron..

[2] Sugioka K., Cheng Y. (2014). Ultrafast lasers—Reliable tools for advanced materials processing. Light Sci. Appl..

[3] Papadopoulos A., Skoulas E., Mimidis A., Perrakis G., Kenanakis G., Tsibidis G.D., Stratakis E. (2019). Biomimetic Omnidirectional Antireflective Glass via Direct Ultrafast Laser Nanostructuring. Adv. Mater..

[4] Krishna H., Favazza C., Gangopadhyay A.K., Kalyanaraman R. (2008). Functional nanostructures through nanosecond laser dewetting of thin metal films. JOM.

[5] Bizi-Bandoki P., Benayoun S., Valette S., Beaugiraud B., Audouard E. (2011). Modifications of roughness and wettability properties of metals induced by femtosecond laser treatment. Appl. Surf. Sci..

[6] Woloszynski T., Touche T., Podsiadlo P., Stachowiak G.W., Cayer-Barrioz J., Mazuyer D. (2019). Effects of Nanoscale Ripple Texture on Friction and Film Thickness in EHL Contacts. Tribol. Lett..

[7] Rudenko A., Colombier J.-P., Höhm S., Rosenfeld A., Krüger J., Bonse J., Itina T.E. (2017). Spontaneous periodic ordering on the surface and in the bulk of dielectrics irradiated by ultrafast laser: A shared electromagnetic origin. Sci. Rep..

[8] Bonse J., Krüger J., Höhm S., Rosenfeld A. (2012). Femtosecond laser-induced periodic surface structures. J. Laser Appl..

[9] Rudenko A., Abou-Saleh A., Pigeon F., Mauclair C., Garrelie F., Stoian R., Colombier J.P. (2020). High-frequency periodic patterns driven by non-radiative fields coupled with Marangoni convection instabilities on laser-excited metal surfaces. Acta Mater..

[10] Skolski J.Z.P., Römer G.R.B.E., Vincenc Obona J., Huis in ’t Veld A.J. (2014). Modeling laser-induced periodic surface structures: Finite-difference time-domain feedback simulations. J. Appl. Phys..

[11] Tsibidis G.D., Fotakis C., Stratakis E. (2015). From ripples to spikes: A hydrodynamical mechanism to interpret femtosecond laser-induced self-assembled structures. Phys. Rev. B.

[12] Giannuzzi G., Gaudiuso C., Franco C.D., Scamarcio G., Lugarà P.M., Ancona A. (2019). Large area laser-induced periodic surface structures on steel by bursts of femtosecond pulses with picosecond delays. Opt. Lasers Eng..

[13] Colombier J.-P., Rudenko A., Silaeva E., Zhang H., Sedao X., Bévillon E., Reynaud S., Maurice C., Pigeon F., Garrelie F. (2020). Mixing periodic topographies and structural patterns on silicon surfaces mediated by ultrafast photoexcited charge carriers. Phys. Rev. Res..

[14] Abou Saleh A., Rudenko A., Reynaud S., Pigeon F., Garrelie F., Colombier J.-P. (2020). Sub-100 nm 2D nanopatterning on a large scale by ultrafast laser energy regulation. Nanoscale.

[15] Gnilitskyi I., Derrien T.J.-Y., Levy Y., Bulgakova N.M., Mocek T., Orazi L. (2017). High-speed manufacturing of highly regular femtosecond laser-induced periodic surface structures: Physical origin of regularity. Sci. Rep..

[16] Li C., Cheng G., Sedao X., Zhang W., Zhang H., Faure N., Jamon D., Colombier J.-P., Stoian R. (2016). Scattering effects and high-spatial-frequency nanostructures on ultrafast laser irradiated surfaces of zirconium metallic alloys with nano-scaled topographies. Opt. Express.

[17] Ran L., Qu S. Femtosecond laser induced surface structures on amorphous alloys. Proceedings of the 2011 Academic International Symposium on Optoelectronics and Microelectronics Technology.

[18] Hoppius J.S., Bialuschewski D., Mathur S., Ostendorf A., Gurevich E.L. (2018). Femtosecond laser crystallization of amorphous titanium oxide thin films. Appl. Phys. Lett..

[19] Ma F., Yang J., Zhu X., Liang C., Wang H. (2010). Femtosecond laser-induced concentric ring microstructures on Zr-based metallic glass. Appl. Surf. Sci..

[20] Apreutesei M., Steyer P., Joly-Pottuz L., Billard A., Qiao J., Cardinal S., Sanchette F., Pelletier J.M., Esnouf C. (2014). Microstructural, thermal and mechanical behavior of co-sputtered binary Zr–Cu thin film metallic glasses. Thin Solid Film..

[21] Apreutesei M., Steyer P., Billard A., Joly-Pottuz L., Esnouf C. (2015). Zr–Cu thin film metallic glasses: An assessment of the thermal stability and phases’ transformation mechanisms. J. Alloys Compd..

[22] Liu J.M. (1982). Simple technique for measurements of pulsed Gaussian-beam spot sizes. Opt. Lett..

[23] Abou-Saleh A., Karim E.T., Maurice C., Reynaud S., Pigeon F., Garrelie F., Zhigilei L.V., Colombier J.P. (2018). Spallation-induced roughness promoting high spatial frequency nanostructure formation on Cr. Appl. Phys. A.

[24] Aguilar A., Mauclair C., Faure N., Colombier J.-P., Stoian R. (2017). In-situ high-resolution visualization of laser-induced periodic nanostructures driven by optical feedback. Sci. Rep..

[25] Zhang W., Cheng G., Hui X.D., Feng Q. (2014). Abnormal ripple patterns with enhanced regularity and continuity in a bulk metallic glass induced by femtosecond laser irradiation. Appl. Phys. A.

[26] Lei Y., Yang J., Cong C., Guo C. (2020). Fabrication of homogenous subwavelength grating structures on metallic glass using double-pulsed femtosecond lasers. Opt. Lasers Eng..

[27] Etiemble A., Der Loughian C., Apreutesei M., Langlois C., Cardinal S., Pelletier J.M., Pierson J.-F., Steyer P. (2017). Innovative Zr-Cu-Ag thin film metallic glass deposed by magnetron PVD sputtering for antibacterial applications. J. Alloys Compd..

[28] Žemaitis A., Mimidis A., Papadopoulos A., Gečys P., Račiukaitis G., Stratakis E., Gedvilas M. (2020). Controlling the wettability of stainless steel from highly-hydrophilic to super-hydrophobic by femtosecond laser-induced ripples and nanospikes. RSC Adv..

[29] Liu Y.-H., Yeh S.-C., Cheng C.-W. (2020). Two-Dimensional Periodic Nanostructure Fabricated on Titanium by Femtosecond Green Laser. Nanomaterials.

[30] Bonse J., Baudach S., Krüger J., Kautek W., Lenzner M. (2002). Femtosecond laser ablation of silicon–modification thresholds and morphology. Appl. Phys. A.

[31] Buividas R., Mikutis M., Juodkazis S. (2014). Surface and bulk structuring of materials by ripples with long and short laser pulses: Recent advances. Prog. Quantum Electron..

[32] Zhang H., Colombier J.-P., Li C., Faure N., Cheng G., Stoian R. (2015). Coherence in ultrafast laser-induced periodic surface structures. Phys. Rev. B.

[33] Rudenko A., Mauclair C., Garrelie F., Stoian R., Colombier J.-P. (2019). Self-organization of surfaces on the nanoscale by topography-mediated selection of quasi-cylindrical and plasmonic waves. Nanophotonics.

[34] Sipe J.E., Young J.F., Preston J.S., van Driel H.M. (1983). Laser-induced periodic surface structure. I. Theory. Phys. Rev. B.

[35] Young J.F., Preston J.S., van Driel H.M., Sipe J.E. (1983). Laser-induced periodic surface structure. II. Experiments on Ge, Si, Al, and brass. Phys. Rev. B.

[36] Lopez-Santos C., Puerto D., Siegel J., Macias-Montero M., Florian C., Gil-Rostra J., López-Flores V., Borras A., González-Elipe A.R., Solis J. (2021). Anisotropic Resistivity Surfaces Produced in ITO Films by Laser-Induced Nanoscale Self-organization. Adv. Opt. Mater..

[37] Han X.J., Schober H.R. (2011). Transport properties and Stokes-Einstein relation in a computer-simulated glass-forming Cu_33.3_Zr_66__.7_ melt. Phys. Rev. B.

